# Pulse wave velocity and neutrophil gelatinase-associated lipocalin as predictors of acute kidney injury following aortic valve replacement

**DOI:** 10.1186/1749-8090-9-89

**Published:** 2014-05-17

**Authors:** Emaddin Kidher, Leanne Harling, Hutan Ashrafian, Hatam Naase, Andrew Chukwuemeka, Jon Anderson, Darrel P Francis, Thanos Athanasiou

**Affiliations:** 1 The Department of Surgery and Cancer, Imperial College London, St Mary’s Hospital, 10th Floor, QEQM Wing, St Mary’s Hospital, Praed Street, London W2 1NY, UK; 2 Imperial College Healthcare NHS Trust, Hammersmith Hospital, London, UK; 3 International Centre for Circulatory Health, Imperial College Healthcare NHS Trust, St Mary’s Hospital, London, UK

**Keywords:** Pulse wave velocity, Aortic stiffness, PWV, Neutrophil gelatinase-associated lipocalin, NGAL, Acute kidney injury, AKI, Aortic valve replacement, AVR

## Abstract

**Background:**

Accurate prediction, early detection and treatment of acute kidney injury (AKI) are essential for improving post-operative outcomes. This study aimed to examine the role of aortic stiffness and neutrophil gelatinase-associated lipocalin (NGAL) as predictors of AKI or need for early medical renal intervention following aortic valve replacement (AVR).

**Methods:**

Aortic pulse wave velocity and plasma NGAL were measured pre-operatively in recruited patients undergoing AVR for aortic stenosis (AS). Plasma NGAL was also measured at 3 and 18–24 hours after cardiopulmonary bypass (CPB). AKI was defined using RIFLE criteria. Early medical renal intervention included diuretics or dopamine infusion exclusively for renal causes.

**Results:**

Fifty-three patients aged 71 ± 9 years were included. Sixteen (30%) developed AKI (AKI-Yes) and 24 patients (45%) received early medical intervention (Intervention-Yes). There was no significant difference in the demographic, clinical or operative characteristics between the two groups for either outcome. PWV did not significantly correlate with AKI (*r* = 0.12, *P* = 0.13) or early intervention (*r* = 0.18, *P* = 0.18). At 3 h post-CPB, plasma NGAL was a much stronger predictor of both AKI and the need for early medical intervention than conventional markers such as creatinine (AKI: AUC 83%, 95% CI 0.70–0.95 vs. AUC 65%, 95% CI 0.47- 0.82; Medical intervention: AUC 84%, 95% CI 0.72–0.96 vs. AUC 56%, 95% CI 0.38–0.73). Post-CPB (3 hr) plasma NGAL was also significantly associated with AKI (*r* = 0.68, *P* < 0.001) at levels above 150 ng/ml; and significantly associated with early intervention (*r* = 0.64, *P* < 0.001) above 136 ng/ml. Simple linear regression showed no relationship between PWV and NGAL levels.

**Conclusion:**

Aortic PWV does not correlate significantly with post-operative AKI or plasma NGAL levels in surgical AS patients. Post-operative NGAL is however an early and powerful predictive biomarker of both post-operative AKI and the need for early medical renal intervention and should consequently be considered in prediction models for AKI after cardiac surgery.

## Background

Post-cardiac surgery acute kidney injury (AKI) is associated with significant morbidity and mortality [1,2], and may occur in up to 30% of adult patients [3,4], with between 1% and 5% requiring renal replacement therapy [5-7]. As such, early recognition and, where possible, prevention of AKI is of paramount importance and continues to be the subject of extensive clinical and basic science research.

Post cardiac-surgical AKI is a complex and multifactorial process that is not fully understood. At least six major injury pathways have been identified including exogenous and endogenous toxins, metabolic factors, ischemia and reperfusion, neurohormonal activation, inflammation, and oxidative stress [8]. Several large clinical studies have been used to develop risk stratification models to achieve better AKI prediction, prevention and management [3], and a long list of predictors/risk factors have been identified [3,9]. As renal perfusion and subsequent kidney function may also be affected by abnormal blood flow patterns, in chronic kidney disease (CKD), non-invasive markers such as aortic stiffness have also been used as predictive and prognostic markers of renal function [10,11]. There is limited evidence to link aortic stiffness with renal function in non-kidney disease patients [12-14], and its role after cardiac surgery is yet to be investigated. However, preliminary data does suggest that elevated arterial stiffness may be an early predictor of renal function decline in the general population [12,13].

Acute Kidney Injury (AKI) is most commonly defined by three quantitative measures including serum creatinine, glomerular filtration rate and urine output. Serum creatinine is currently the gold standard biomarker of renal function, however its application is limited by the 12–48 hour latency period between AKI onset and its elevation in the serum. As such, novel troponin-like biomarkers of AKI including cystatin C, interleukins (IL), kidney injury molecule 1 (KIM 1), C-reactive protein (CRP), and neutrophil gelatinase-associated lipocalin (NGAL) have been extensively investigated [15].

NGAL is a 25 kDa lipocalin expressed in human neutrophils and at low levels in several other human tissues including the kidneys, trachea, lungs, and colon [16,17]. The NGAL concentration in renal proximal tubules increases rapidly after renal ischemia–reperfusion injury and, as such, it has been investigated as a novel biomarker for AKI [13,18,19]. In addition, NGAL concentrations begin to increase in serum and urine after 2 hours in children [20] and 1 hour in adults following CPB [21], rendering it a useful biomarker for AKI after cardiac surgery. Indeed, work by Haase-Fielitz and colleagues demonstrated that plasma NGAL levels of >150 ng/ml taken on arrival in the intensive care unit (ITU) had a 76% sensitivity and 91% specificity for predicting AKI [22]. However, the overall results of studies investigating plasma NGAL as a predictor of AKI after cardiac surgery are inconsistent, with variations in patient population, procedure type and sample collection timing limiting its current uptake in clinical practice [15,23]. Furthermore, no studies have yet evaluated the relationship between plasma NGAL and aortic stiffness as predictors of AKI after cardiac surgery.

The objectives of this study are therefore to assess: (a) if aortic stiffness (measured by pulse wave velocity (PWV)) is a predictor of post- aortic valve replacement (AVR) AKI or the need for early medical renal intervention; (b) if NGAL may be a predictor of post-AVR AKI or predict the need for early medical renal intervention; and (c) if PWV is related to NGAL.

## Methods

### Overview

Ethical approval (11/H0709/3) and participant informed consent were obtained prior to undertaking this study. Patients undergoing AVR for moderate to severe AS between June 2010 and August 2012 were eligible. Patients were excluded if they had/presented with: aortic dissection; moderate or severe aortic regurgitation; aortic aneurysm; Marfan’s syndrome; known case of chronic kidney disease. All data were recorded prospectively.

Carotid-femoral PWV was measured pre-AVR. Markers being studied (serum creatinine, GFR, and NGAL) were measured from blood samples collected at three time-points: Pre-operatively, 3 hours post-CPB, and 18–24 hours post-CPB. In addition, creatinine was measured daily until not indicated post-operatively to assess AKI development and/or progression.

### Pulse wave velocity measurement protocol

Prior to measurement all participants abstained from tobacco, alcohol and tea or coffee for at least 2 hours. Patients were rested for 10 minutes in a quiet, temperature-controlled room (22-25C°) before the hemodynamic measurements were taken. A digital sphygmomanometer (Criticare, Model 506 N3, Waukesha, USA) was used to measure brachial blood pressure (BP) and heart rate. BP measurements were taken before and after PWV assessment and the mean used for analysis. Aortic PWV (carotid-femoral PWV) was measuered with an automatic applanation tonometry system (SphygmoCor Vx, AtCor Medical, Australia). Briefly, with the patient resting in a supine position, ECG-gated pulse waveforms were obtained sequentially from the common carotid and then femoral arteries. Propagation time of the pulse wave was measured from the foot of the carotid waveform to that of the femoral waveform, referenced to the R wave on the recorded ECG. The transit distance (mm) was measured over the body surface by subtracting the supra-sternal notch to carotid site distance from the supra-sternal notch to femoral site distance. PWV was then calculated in m/s using the Sphygmocor device by dividing the travelled distance by the propagation time. Three to five readings were obtained per patient and PWV determined by averaging the measurements meeting quality control parameters outlined by SphygmoCor.

### Clinical assessment

A full pre-operative medical history was recorded. Post-operative hourly urine output was recorded for the first 36–48 hours, and for longer periods if the patient required further renal attention or intervention. The use of diuretics or dopamine as preventive renal therapy prior to onset of AKI was also recorded. Fluid balance at the end of day zero and day one post-operatively, the duration of continuous intravenous fluid (IVF) infusion, and the need for any blood product post-operatively were also recorded for each patient.

### Serum creatinine and glomerular filtration rate

All serum creatinine and GFR measurements were determined by laboratory staff blinded to the study parameters. The decision to use the routine clinical laboratory was made to accurately reflect a real clinical setting for identifying AKI patients. The GFR was calculated using the Modification of Diet in Renal Disease (MDRD) equation of GFR:

$$\begin{array}{l} =186\;x\;{\left(\mathrm{Serum}\;\mathrm{Creatinine}/88.4\right)}^{-1.154}\;x\;{\left(\mathrm{Age}\right)}^{-0.203}\\ \;\;x\;\left(0.742\;\mathrm{if}\;\mathrm{female}\right)\;x\;\left(1.210\;\mathrm{if}\;\mathrm{black}\right) \end{array}$$

### Neutrophil gelatinase-associated lipocalin (NGAL)

NGAL was measured pre-operatively, 3 h post-CPB, and on day one (18–24 h post-CPB) after AVR [22,24-26]. NGAL levels were measured using a point-of-care, fluorescence-detected immunoassay test kit (Biosite Triage, Biosite Incorporated, San Diego, CA, USA currently Alere Triage MeterPro, Alere Ltd., Stockport, UK), validated against enzyme-linked immunosorbent assay (ELISA) as previously described [24,27]. Ethylenediaminetetraacetic acid (EDTA)-containing tubes were used to collect the blood samples through venipuncture pre-operatively and through the central venous line post-operatively. Briefly, 250 μL of plasma was added to the disposal test strip that has a filter to separate cells and any other particles allowing only plasma to reach the reaction chamber that contains NGAL-specific monoclonal antibody conjugated to a fluorescent nanoparticle, NGAL antigen immobilized on a solid phase, and stabilizers. After the plasma forms a fluorescent antibody conjugate with the detection nanoparticles, it flows down the diagnostic lane via capillary action. NGAL present in the specimen prevents binding of the fluorescent detection particles to the solid phase immobilized in the detection zone, so that the analyte concentration is inversely proportional to the fluorescence-detected [24]. The test strip is then inserted into the Triage machine, which reports NGAL level. The entire measurement procedure takes approximately 15 minutes to complete. All NGAL measurements were performed within 30 minutes of sample collection after standard machine calibration with a standard calibrating strip. The triage assay has a detection range of 60 to 1300 ng/ml with a coefficient of variation of 10% to 15%. All tests were performed by a single operator (EK) after training from the manufacturer.

### Acute kidney injury

The primary outcome was the development of AKI, as defined by the RIFLE criteria (Table 1) [28]. In addition, staging of chronic kidney disease was recorded according to established GFR cut-offs [29].

**Table 1:** RIFLE criteria for acute kidney injury

**Criterion**	**Threshold for cut-off**
**Risk**	***Serum Creatinine:*** 1.5-fold increase
	***GFR:*** 25% decrease
	***Urine Output:*** less than 0.5 ml/kg/hour for 6 hours
**Injury**	***Serum Creatinine:*** 2-fold increase
	***GFR:*** 50% decrease
	***Urine Output:*** less than 0.5 ml/kg/hour for 12 hours
**Failure**	***Serum Creatinine:*** 3-fold increase
	***GFR:*** 70% decrease
	***Urine Output:*** less than 0.3 ml/kg/hour for 24 hours or anuria for 12 hours
**Loss**	Complete loss of kidney function for more than 4 weeks
**ESRD**	Complete loss of kidney function for more than 3 months

Clinical intervention for renal dysfunction often precedes defined cut-offs for AKI. Our institutional practice is therefore to instigate early medical intervention where urine output is less than 0.5 ml/kg/hour for 3 hours. Example interventions include IV diuretics (repeated doses over 24–48 hours) and low dose IV dopamine infusion. Routine clinical practices were maintained for patients enrolled in this study in order to maintain blinding of clinical staff and avoid bias. However, additional subgroup analysis was performed according to IV diuretic or dopamine use in order to account for these practices.

### Statistical analysis

Statistical analysis was conducted using IBM SPSS 20.0 software package (IBM Corporation, Armonk, NY, USA). Patient characteristics and results are expressed as medians or means ± standard deviation for continuous variables and as frequencies for categorical variables. Two tailed t-tests were considered significant at the 0.05 level. Test for normality was carried out on all relevant variables.

Comparative analyses between the two AKI groups (presence of AKI [AKI-Yes] and absence of AKI [AKI-No]) and the two early medical renal intervention groups (required intervention [Intervention-Yes] and did not require intervention [Intervention-No]) were carried out using independent samples *t*-tests or non-parametric equivalent (Mann–Whitney *U* test) for continuous variables, and Pearson Chi-square or Fisher’s exact for categorical variables. Correlation analysis between variables was conducted using Spearman’s rank-order correlation and point biserial correlation (for dichotomous variables). To assess the diagnostic utility of NGAL measurements for predicting primary and secondary outcomes (post-operative AKI and the need for early medical renal intervention) compared to serum creatinine, conventional receiver operating characteristic (ROC) curves were produced and the area under the curve (AUC) calculated for each test. A 3 h post-CPB NGAL cut-off point was selected based on best sensitivity and specificity values. The association between plasma NGAL level equal or greater than the selected cut-off point and the development of primary and secondary outcomes was then tested using Contingency Table Analysis and Odds Ratios (ORs). The association between increasing levels of plasma NGAL and the development of primary and secondary outcomes was determined by dividing the population into quartiles according to NGAL level and calculated the percentage of each outcome for each quartile.

The study was approved by the North London Research Ethics Committee 3 (11/H0709/3).

## Results

### Descriptive results

Fifty-three patients were included in this study, including 37 (70%) males with a mean age of 71 ± 9 years (Table 2). None had a history of pre-operative renal disease or were in an emergent or unstable state pre-operatively. Sixteen patients (30%) developed AKI according to the RIFLE criteria, and 24 patients (45%) had early medical intervention (Intervention-Yes), indicated by low urine output for less than 6 hours. Pre-operatively, 89% of patients had stage 1 or 2 CKD, 11% had stage 3, and no patient had stage 4 or 5 disease. There was no significant difference between AKI-Yes and AKI-No or between Intervention-Yes and Intervention-No groups with respect to age, gender, hemodynamic parameters, or main clinical and operative characteristics as shown in Table 2.

**Table 2:** Demographic and clinical characteristics

**Parameter**	**Total**	**AKI-No**	**AKI-Yes**	***P*-value**	**Intervention-No**	**Intervention-Yes**	***P*-value**
	**n = 53**	**n = 37**	**n = 16**		**n = 29**	**n = 24**	
Male (n [%])	37 (70%)	25 (67%)	12 (75%)	0.58	22 (76%)	15 (62%)	0.29
Age (years)	71 ± 9	70 ± 9	73 ± 8	0.39	69 ± 8	73 ± 9	0.14
White Caucasian (n [%])	52 (98%)	37 (100%)	15 (93%)	0.30	29 (100%)	23 (96%)	0.45
SBP (mmHg)	138 ± 17	136 ± 17	143 ± 18	0.11	139 ± 16	136 ± 20	0.68
DBP (mmHg)	76 ± 12	76 ± 12	77 ± 14	0.51	79 ± 10	79 ± 13	0.14
MAP (mmHg)	97 ± 12	96 ± 12	99 ± 12	0.12	99 ± 11	94 ± 13	0.55
PP (mmHg)	62 ± 15	60 ± 13	66 ± 18	0.23	61 ± 13	63 ± 17	0.81
DM (n [%])	8 (16%)	7 (19%)	1 (6%)	0.23	5 (17%)	3 (12%)	0.68
Hypertensive (n [%])	36 (68%)	25 (68%)	11 (69%)	0.93	17 (59%)	19 (79%)	0.11
Dyslipidemia (n [%])	35 (66%)	25 (68%)	10 (63%)	0.72	17 (59%)	18 (75%)	0.21
Smoking (n [%])	2 (4%)	1 (3%)	1 (6%)	0.25	0 (0.0%)	2 (8%)	0.16
BMI (kg/m^2^)	27.3 ± 4.4	26.6 ± 4.3	28.9 ± 4.4	0.08	27.0 ± 4.4	27.6 ± 4.5	0.56
PVD (n [%])	1 (2%)	0 (0.0%)	1 (6%)	0.30	0 (0.0%)	1 (4%)	0.45
EF	60 ± 15	63 ± 14	55 ± 17	0.16	63 ± 13	57 ± 17	0.33
AVA (cm^2^)	0.74 ± 0.22	0.71 ± 0.21	0.80 ± 0.23	0.24	0.75 ± .22	0.73 ± 0.22	0.69
AVPG (mmHg)	81 ± 24	84 ± 25	73 ± 20	0.12	83 ± 28	78 ± 19	0.55
AVMG (mmHg)	47 ± 12	48 ± 12	44 ± 12	0.31	48 ± 12	47 ± 12	0.78
PWV (m/s)	9.3 ± 2.3	9.1 ± 2.3	9.7 ± 2.1	0.34	8.9 ± 2.1	9.8 ± 2.4	0.13
Logistic EuroScore	5.5 ± 4.3	4.8 ± 3.5	6.9 ± 5.5	0.15	4.2 ± 2.2	6.9 ± 5.6	0.22
Concomitant CABG (n [%])	15 (28%)	10 (27%)	5 (31%)	0.75	9 (30%)	9 (37%)	0.18
Cross clamp time (minutes)	68 ± 20	68 ± 18	67 ± 26	0.66	70 ± 19	65 ± 22	0.17
CPB time (minutes)	87 ± 23	86 ± 22	90 ± 28	0.66	88 ± 23	86 ± 25	0.47
IVF duration (hours)	42 ± 17	42 ± 17	43 ± 17	0.41	38 ± 10	48 ± 22	**0.04**
Fluid balance day 0 (ml)	1987 ± 658	1854 ± 631	2328 ± 622	**0.02**	1948 ± 679	2037 ± 643	0.68
Fluid balance day 1 (ml)	1348 ± 915	1146 ± 854	1853 ± 893	**0.02**	1122 ± 808	1626 ± 978	**0.05**
Post-operative blood products (n [%])	27 (53%)	18 (49%)	9 (56%)	0.51	11 (38%)	16 (66%)	**0.03**
Hospital stay (days)	6.7 ± 1.7	6.4 ± 1.5	7.3 ± 2.0	0.09	6.3 ± 1.5	7.3 ± 1.8	**0.05**

Values are shown as n (%) for categorical variables and means ± standard deviation for continuous variables; bold font indicates statistical significance. *Abbreviations:* AVA, aortic valve area; AVMG, aortic valve mean gradient; AVPG, aortic valve peak gradient; BMI, body mass index; CABG, coronary artery bypass graft; CPB, cardiopulmonary bypass; DBP, diastolic blood pressure; DM, diabetes mellitus; EF, ejection fraction; IVF, intravenous fluid; MAP, mean arterial blood pressure; PP, pulse pressure; PVD, peripheral vascular disease; PWV, pulse wave velocity; SBP, systolic blood pressure.

### Pulse wave velocity, acute kidney injury, and early medical renal intervention

There was no significant difference in pulse wave velocity (PWV) (m/s) between AKI-No and AKI-Yes groups or between Intervention-No and Intervention-Yes groups (Table 2). Bivariate correlation revealed no association between PWV and AKI development (*r* = 0.12, *P* = 0.13) or between PWV and early intervention (*r* = 0.18, *P* = 0.18). There was a trend towards increasing PWV (m/s) with increasing CKD stage: 8.7 ± 1.7 for stage 1, 9.2 ± 2.4 for stage 2, and 10.8 ± 2.2 for stage 3 (*P* = 0.18; ANOVA). There was no correlation between PWV and renal function; the correlation coefficient between PWV and serum creatinine was 0.18 (not significant) and 0.268 (not significant) between PWV and GFR.

### Neutrophil gelatinase-associated lipocalin (NGAL) and acute kidney injury

**Figure 1:**
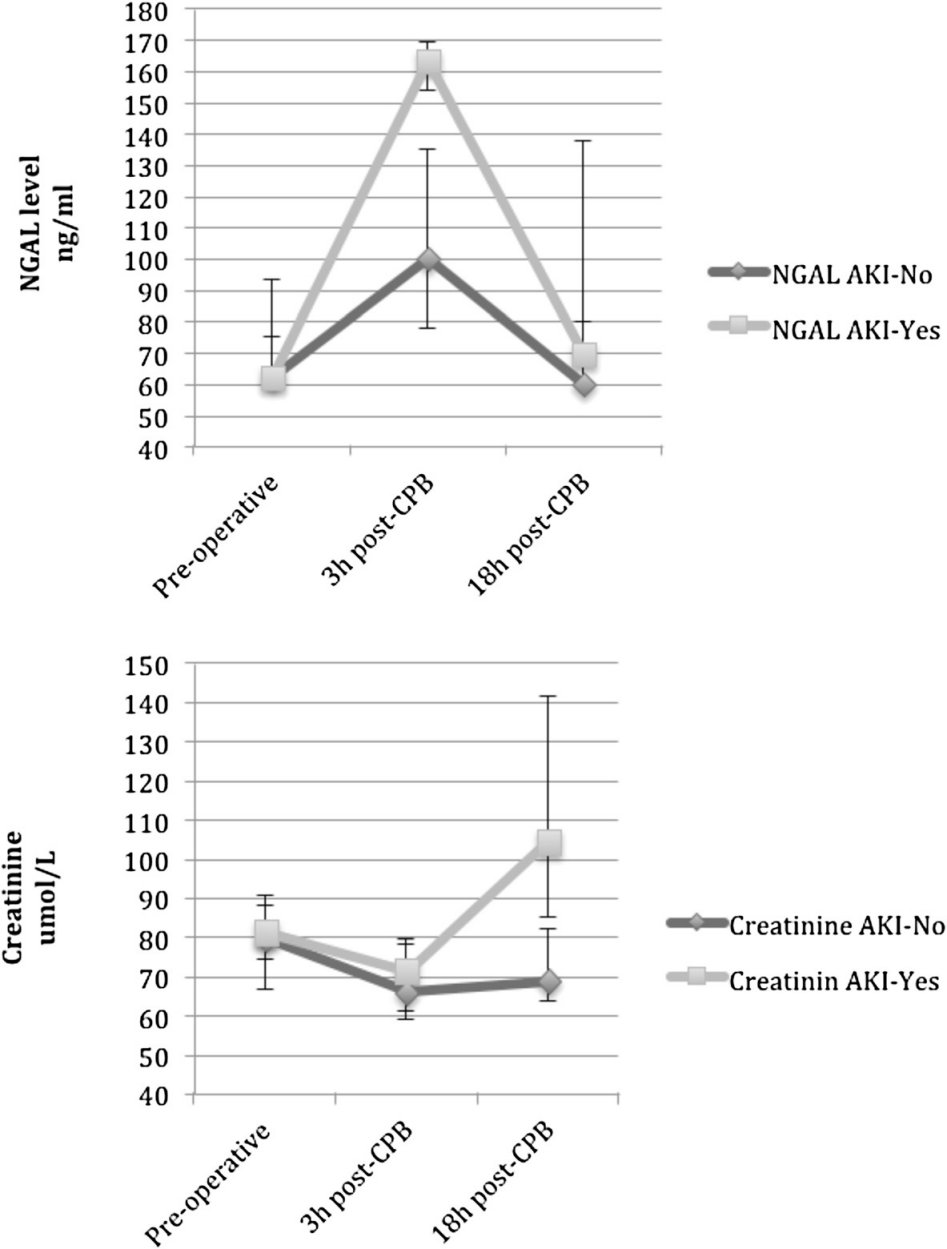
**Trend time of the NGAL and creatinine levels between AKI-No and AKI-Yes groups.** Squares indicate median values; bars represent the first and third quartiles (Q1–Q3). Abbreviations: CPB, cardiopulmonary bypass; NGAL, neutrophil gelatinase-associated lipocalin.

Sixteen patients (30%) developed AKI according to RIFLE criteria, 12 (75%) of which were ‘Risk’ and 4 (25%) ‘Injury’. None progressed to the “failure” stage or required renal replacement therapy. The mean time to AKI development was 34.2 ± 21 hours post-operatively (range, 8–93 hours). AKI was also significantly more frequent in patients with a high BMI and resulted in a significantly longer hospital stay (Table 2). Table 3 summarizes the levels of markers assessed for both AKI groups and over the three time-points; pre-operatively there was no difference in these markers between groups. At the early post-operative stage (3 h post-CPB), NGAL differed significantly (*P* < 0.001) between AKI-No (113 ± 51 ng/ml) and AKI-Yes (167 ± 66 ng/ml) groups (Table 3 and Figure 1). However, at the same 3-hr post CPB timepoints traditional diagnostic markers (creatinine and GFR) were better than baseline pre-operative levels. These traditional diagnostic markers (creatinine and GFR) only became significantly different from baseline 18 hours after CPB, by which time the mean level of NGAL for AKI-Yes group had returned to nearly normal.

**Table 3:** Levels of the renal function markers at different study time-points

**Marker**	**Total**	**AKI-No**	**AKI-Yes**	***P*-value**	**Intervention-No**	**Intervention-Yes**	***P*-value**
	**n = 53**	**n = 37**	**n = 16**		**n = 29**	**n = 24**	
**Creatinine (μmol/L)**							
Pre-operative	80 ± 14	79 ± 14	82 ± 12	0.25	76 ± 11	85 ± 14	**0.02**
3 h post-CPB	71 ± 15	68 ± 13	77 ± 19	0.12	69 ± 13	73 ± 18	0.44
18 h post-CPB	87 ± 35	74 ± 17	116 ± 45	**<0.001**	73 ± 16	103 ± 43	**<0.01**
**GFR (ml/min/1.73 m**^**2**^**)**							
Pre-operative	80 ± 17	80 ± 19	77 ± 13	0.55	86 ± 16	72 ± 15	**<0.01**
3 h post-CPB	94 ± 19	96 ± 19	88 ± 21	0.32	97 ± 18	89 ± 21	0.22
18 h post-CPB	79 ± 26	88 ± 21	58 ± 24	**<0.001**	91 ± 19	65 ± 26	**<0.001**
**NGAL (ng/ml)**							
Pre-operative	80 ± 35	83 ± 40	73 ± 20	0.55	83 ± 40	76 ± 25	0.96
3 h post-CPB	129 ± 61	113 ± 51	167 ± 66	**<0.001**	100 ± 29	164 ± 70	**<0.001**
18 h post-CPB	81 ± 38	74 ± 28	98 ± 53	**0.02**	71 ± 22	94 ± 49	**0.01**

Values are shown as mean ± standard deviation; bold font indicates significant values. *Abbreviations:* CPB, cardiopulmonary bypass; GFR, glomerular filtration rate (Modification of Diet in Renal Disease equation); NGAL, neutrophil gelatinase-associated lipocalin.

### Receiver operating characteristics analysis

**Figure 2:**
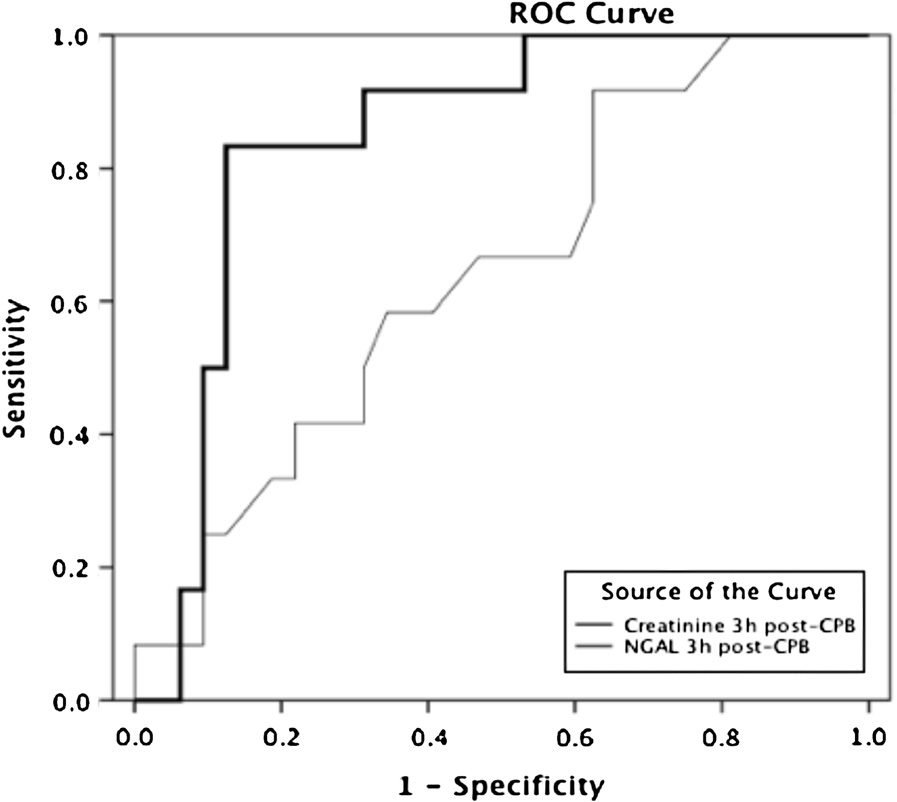
**Receiver operator characteristic (ROC) curve for 3 h post-CPB NGAL and creatinine for the diagnosis of AKI within the next 1–3 days.** The area under the ROC for the NGAL was 0.83 (95% CI 0.70–0.95), indicating a good performance for the diagnosis of AKI, while for creatinine it was 0.65 (95% CI 0.47–0.82). Abbreviations: ROC, receiver operator characteristic; CPB, cardiopulmonary bypass; NGAL, neutrophil gelatinase-associated lipocalin; AKI, acute kidney injury; CI, confidence interval. Diagonal segments are produced by ties.

**Figure 3:**
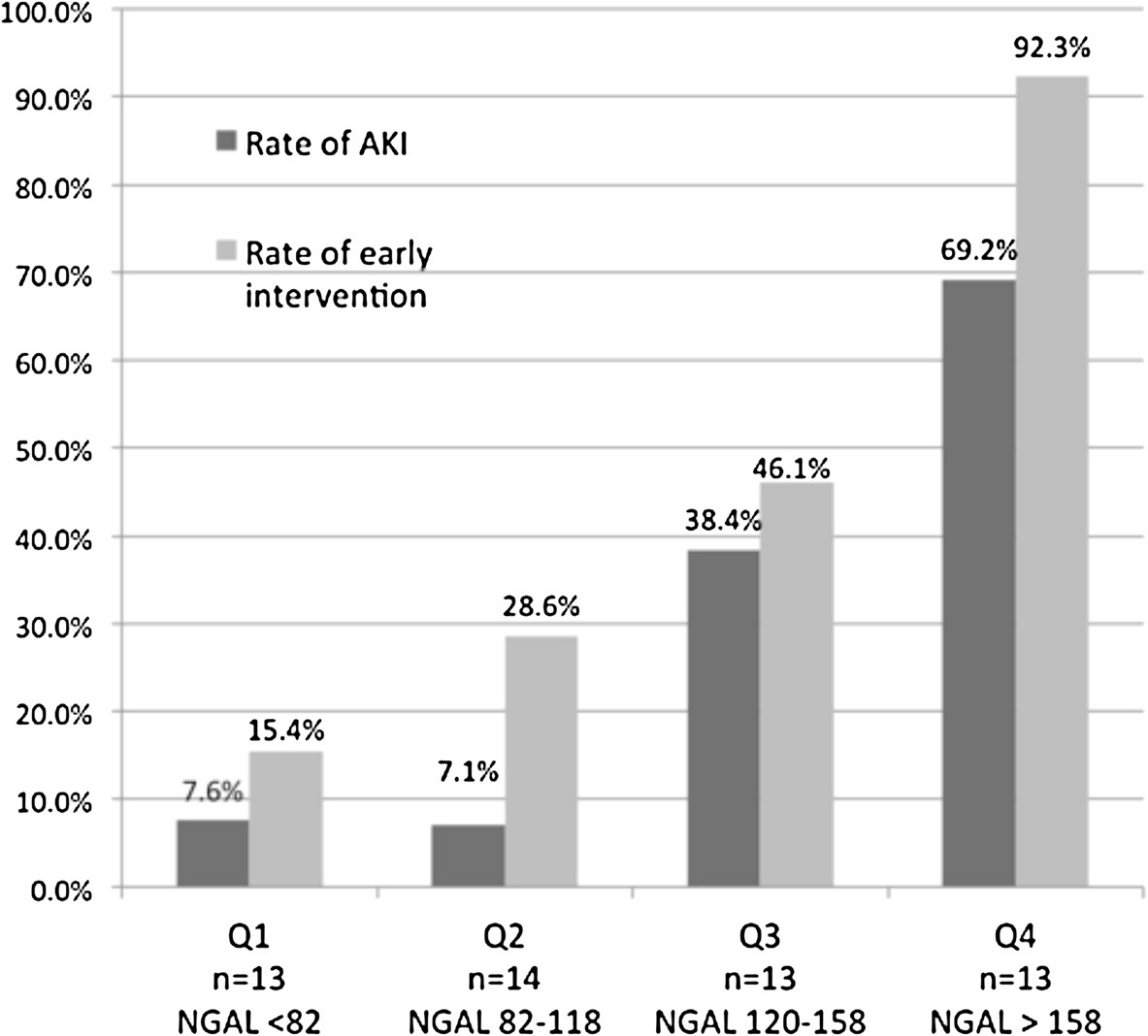
**Rate of AKI and need for early medical renal intervention per quartile for the 3 h post-CPB NGAL level.** There is a marked proportional relationship between increasing NGAL levels (ng/mL) and the rate of AKI and the need for early medical renal intervention. Abbreviations: CPB, cardiopulmonary bypass; NGAL, neutrophil gelatinase-associated lipocalin; AKI, acute kidney injury; Q, quartile.

NGAL taken at 3 hours post-CPB was a much better predictor of AKI than creatinine taken at the same time-point (0.83, 95% confidence interval [CI] 0.70–0.95 vs. 0.65, 95% CI 0.47–0.82, respectively; Figure 2). At a cut-off of 150 ng/ml, 3 h post-CPB NGAL showed an 80% sensitivity and 88.9% specificity for detecting AKI. Furthermore, 3 h post-CPB plasma NGAL ≥ 150 ng/ml was significantly associated with AKI development (Phi-coefficient = 0.68, *P* < 0.001; OR 33.0; 95% CI 6.4–169.5). Dividing the population into quartiles according to 3 h post-CPB NGAL level and calculating the percentage of AKI for each quartile revealed a proportional relationship between increasing NGAL level and the rate of AKI, moving from 7.6% in quartile 1 to 69.2% in quartile 4 (Figure 3).

### NGAL and the need for early medical renal intervention

Twenty-four patients (45%) had early medical intervention (Intervention-Yes group) in an attempt to prevent AKI. Of these, 9 (37.5%) did not develop any RIFLE criteria however 11 developed ‘Risk’ criteria and 4 ‘Injury’ criteria. The 15 patients receiving early medical intervention going on to develop AKI represent 94% of the total AKI patients (n = 16). There were no differences in age, gender, hemodynamic parameters, medical history, or operative characteristics between early medical intervention and no early medical intervention groups (Table 2). However, the intervention-No group had significantly better pre-operative creatinine (76 ± 11 vs. 85 ± 14 μmol/L, respectively; *P* = 0.02) and GFR (86 ± 16 vs 72 ± 15, *P* < 0.01) when compared to the Intervention-Yes group (Table 3). Compared to the Intervention-No group, the Intervention-Yes group had a significantly longer duration of intravenous fluid administration (48 ± 22 hours vs. 38 ± 10 hours, respectively; *P* = 0.04) and higher day one positive fluid balance post-operatively (1626 ± 978 ml vs. 1122 ± 808 ml, respectively; *P* = 0.05; Table 2). Furthermore, intervention-yes patients received significantly more blood products post operatively (16 [66%] Intervention-yes vs. 11 [38%] Intervention-no; *P* = 0.03).

Bivariate correlation analysis showed a significant correlation between early medical intervention and both pre-operative creatinine (r = 0.31; *P* < 0.05) and GFR (r = −0.37; *P* < 0.01). Furthermore, diagnostic accuracy analysis revealed a 68% predictive power for pre-operative creatinine (95% CI 0.53–0.82) and 71% for pre-operative GFR (95% CI 0.57–0.85) in detecting the need for early medical intervention.

Post-operatively, only 3 h post-CPB NGAL was significantly higher in the Intervention-Yes group compared to the Intervention-No group (164 ± 70 vs. 100 ± 29, *P* < 0.001). Furthermore, at a cut-off of 136 ng/ml, 3 h post-CPB NGAL demonstrated a 73.9% sensitivity and 89.3% specificity in detecting the need for early medical intervention, and a predictive power (AUC-ROC) of 84% (95% CI 0.72–0.96). Notably, when calculated for creatinine, the AUC was only 56% (95% CI 0.38–0.73). Plasma NGAL level (3 h post-CPB) equal or greater than 136 ng/ml was associated with a need for early medical renal intervention (Phi-coefficient = 0.64, *P* < 0.001; OR = 23.6 [95% CI 5.1–107.5]). Quartiles of plasma NGAL levels (3 h post-CPB) were proportionally associated with the rate of need for early medical renal intervention moving from 15.4% in Q1 to 92.3% in Q4 (Figure 3).

### NGAL and PWV

Bivariate correlation revealed a clinically significant association between PWV and pre-operative NGAL level (*r* = 0.25, *P* = 0.07), which was lost at 3 h post-CPB (*r* = 0.16, *P* = 0.25). However, this association again became significant at 18 h post-CPB (*r* = 0.30, *P* = 0.03). Simple linear regression showed no relationship between PWV and pre-operative NGAL level (Beta = 0.23, *P* = 0.1), 3 h post-CPB NGAL level (Beta = 0.09, *P* = 0.51), or 18 h post-CPB NGAL level (Beta = 0.11, *P* = 0.41).

## Discussion

### The role of PWV as a marker of acute kidney injury

The results of this study demonstrate a non-significant increase in the pre-operative aortic stiffness (as measured by PWV) of patients developing AKI or requiring early renal intervention after aortic valve surgery. Similarly, we observe a trend towards increasing PWV with increasing severity of kidney disease (taken stepwise from CKD stages 1–5) although this did not reach statistical significance.

These results appear to conflict previous studies which link increasing aortic stiffness to reduced renal function [11,30]. However, on closer scrutiny these studies have been conducted on patients with moderate to severe reductions in GFR (40 to 65 ml/min/1.73 m^2^), whereas our study examines patients with normal or only mild pre-operative renal impairment (mean GFR of 80 ± 17 ml/min/1.73 m^2^). Similarly, when examining studies demonstrating a significant stepwise increase in PWV with CKD stage [11,30,31], these changes in PWV are found to be most significant when comparing CKD stages 1 and 2 to more advanced stages of CKD [31]. Nakagawa et al. did observe similar findings when comparing PWV in patients with stage 1 and stage 2 disease [30], however this observation was made with a sample number of 409 patients (17 stage 1; 392 stage 2), significantly larger than the numbers studied here. As such, we can conclude from our results that at normal to mild levels of pre-operative renal impairment, PWV correlates only weakly with post-operative acute kidney injury, the severity of kidney disease and the need for early renal intervention. At more severe levels of renal dysfunction (CKD stage 3–5) previously reported evidence suggests there may be a much stronger inverse correlation with PWV, although this could not be verified in this study owing to insufficient patient numbers in these groups.

### NGAL as a marker of AKI

Our results demonstrate a significant positive correlation between 3 h post-CPB NGAL levels and both post-AVR AKI and the need for early renal intervention. Furthermore, these findings appear more reliable at predicting post-operative kidney dysfunction than pre-operative or early post-operative creatinine and/or GFR. Indeed, in our study 94% of patients who went on to develop AKI had been previously flagged by clinicians as requiring early preventative medical therapy based on a low urine output. This early preventive medical intervention was initiated despite creatinine and GFR levels falling within the normal range, highlighting the need for an early biomarker of AKI above and beyond those currently available.

Notably, by 18 hours post-CPB, mean NGAL had returned to pre-operative levels even in the AKI-yes group, signifying renal recovery just at the point when creatinine and GFR measurements have begun to register as abnormal. This is highlighted in the 83% predictive power of 3 hr post-CPB NGAL to detect AKI, which is comparable to the 86% predictive power of 18 hr creatinine. Indeed, no patients in the AKI-Yes group progressed to RIFLE stage “failure” or required renal replacement therapy.

The number of studies currently investigating the predictive value of plasma NGAL in cardiac patients is limited, and none explore its predictive value for the need for early medical renal intervention [15]. Our results corroborate findings by Tuladhar et al. [25]. and Haase-Fielitz et al [22],. who show significant positive correlation between early post-CPB NGAL and AKI. We also demonstrate 3 hr post-CPB NGAL to have 80% sensitivity and 88.9% specificity for detecting AKI at a cut-off of 150 ng/ml, again similar to the results of Haase-Fielitz and colleagues [22]. In addition, we demonstrate that plasma NGAL may also be a highly sensitive (73.9%) and specific (89.3%) biomarker for detecting the need for early medical intervention at a slightly lower cut-off of 136 ng/ml. As such, we believe that plasma NGAL taken 3 hours after CPB may be a robust and reliable biomarker of both post-operative acute kidney injury and the need for early intervention in cardiac surgical patients. Moreover, NGAL was the only biomarker to differ significantly between patients requiring early renal intervention and those that did not (164 ± 70 vs. 100 ± 29, *P* < 0.001), outperforming conventional markers such as GFR and creatinine.

### The relationship between PWV and NGAL

Pulse wave velocity was not found to be significantly associated with plasma NGAL levels in our patient cohort, as also evidenced by the absence of association between PWV and AKI. Again, it is possible that this is in part due to the overall good level of baseline renal function in our patient cohort, and that at lower levels of baseline renal function, aortic stiffness may indeed correlate with AKI and subsequently NGAL. Further research is therefore required at more severe levels of baseline renal dysfunction (CKD stage 3–5), to determine whether any correlation does indeed exist between PWV, AKI and NGAL in these patients.

### Limitations

The results of this study should be considered in the context of several limitations. First, the majority of our patients had normal to mildly impaired baseline renal function, and as such these findings may not be generalizable to those with moderate to severe baseline renal impairment. Second, our study represents a relatively small sample size, which may be insufficient to predict renal outcomes on the basis of pre-operative criteria. However, the aim of this study was not to identify a list of predictors or to build another AKI risk model; rather, we present a preliminary study to test a novel hypothesis in regards to the relationship between aortic stiffness and AKI.

## Conclusions

Aortic PWV did not correlate significantly with post-operative AKI or plasma NGAL levels in surgical aortic stenosis patients with normal to mildly impaired renal function. However, in order to formulate more concrete conclusions about the association between aortic stiffness and post operative AKI, adequately powered prospective studies are required including larger sample sizes and subgroup analyses of patients with baseline renal function ranging from normal to severely impaired.

Our results do however demonstrate that early post-operative plasma NGAL may act as a sensitive and specific biomarker of both post-operative AKI and the need for early medical renal intervention. Rises in early plasma NGAL may be therefore be used to predict patients at risk of renal dysfunction and allow early administration of preventative therapies to prevent progression to AKI. This may be utilized alongside conventional late stage markers such as creatinine and GFR to allow us to optimize the early recognition and treatment of post-operative renal dysfunction.

## Competing interests

The authors declare that there is no conflict of interests. Alere Ltd., Stockport, UK provided the point-of-care Alere Triage MeterPro and tests strips for NGAL free of charge, but they had no control or influence on the study design, data analysis or publications.

## Authors’ contributions

TA provided the original idea for this study and supervised this research. Patients were recruited and data collected by EK. EK and HN performed samples processing. Data analysis was performed by EK and TA. EK, LH and HA drafted and finalized the manuscript. Cardiology support and additional supervision was provided by DF. All authors read and agreed the final manuscript prior to submission.
